# Analysis of microRNA (miRNA) expression profiles reveals 11 key biomarkers associated with non-small cell lung cancer

**DOI:** 10.1186/s12957-017-1244-y

**Published:** 2017-09-19

**Authors:** Ke Wang, Mingwei Chen, Wei Wu

**Affiliations:** 1 Department of Clinical Medicine, Xi’an Medical University, Shaanxi Research Center of Respiratory Diseases Prevention and Diagnosis and Treatment, Xi’an City, Shaanxi Province 710021 China; 2 Department of Respiratory, First Affiliated Hospital of Xi’an Medical University, Shaanxi Research Center of Respiratory Diseases Prevention & Diagnosis and Treatment, Xi’an City, Shaanxi Province China

**Keywords:** Non-small cell lung cancer, miRNAs, Biomarker, Meta-analysis

## Abstract

**Background:**

Non-small cell lung cancer (NSCLC) accounts for more than 85% of lung cancer cases which cause most of cancer-related deaths globally. However, the results vary largely in different studies due to different platforms and sample sizes. Here, we aim to identify the key miRNAs in the carcinogenesis of NSCLC that might be potential biomarkers for this cancer.

**Methods:**

Meta-analysis was performed on miRNA profile using seven datasets of NSCLC studies. Furthermore, we predicted and investigated the functions of genes regulated by key miRNAs.

**Results:**

Eleven key miRNAs were identified, including 2 significantly upregulated ones (hsa-miR-21-5p and hsa-miR-233-3p) and 9 downregulated ones (hsa-miR-126-3p, hsa-miR-133a-3p, hsa-miR-140-5p, hsa-miR-143-5p, hsa-miR-145-5p, hsa-miR-30a-5p, hsa-miR-30d-3p, hsa-miR-328-3pn, and hsa-miR-451). The functional enrichment analysis revealed that both up- and downregulated miRNAs were proportionally associated with regulation of transcription from RNA polymerase II promoter. According to transcription factor analysis, there were 65 (43.9%) transcription factors influenced by both up- and downregulated miRNAs.

**Conclusions:**

In this study, 11 meta-signature miRNAs, as well as their target genes and transcription factors, were found to play significant role in carcinogenesis of NSCLC. These target genes identified in our study may be profitable to diagnosis and prognostic prediction of NSCLC as biomarkers.

**Electronic supplementary material:**

The online version of this article (10.1186/s12957-017-1244-y) contains supplementary material, which is available to authorized users.

## Background

Although great effort has been put in research and prevention strategies, lung cancer is still the most common cancer globally [1]. To date, it even shows increasing tendency in both annual incidence and mortality [2]. Non-small cell lung cancer (NSCLC) accounts for almost 85–90% of all lung cancers [3]. Additionally, lung cancer remains to be the most common cause giving rise to cancer-related deaths worldwide. In the USA, one 5-year survival rate is shown to be less than 20% in patients [4]. Usually, the occurrence and development of NSCLC results from multistep carcinogenesis, and these involve in various changes of gene expression and signal transduction pathways [5, 6]. Nevertheless, there are still unclear mechanisms, which help to promote carcinogenesis of NSCLC, that need to be exploited. Hence, a search of novel biomarkers for the diagnosis and prognostic prediction of NSCLC is critical for the patients in order to receive proper therapies as early as possible.

MicroRNAs (miRNAs) are a type of small non-coding RNA molecules, which acts in RNA silencing and post-transcriptionally regulating gene expressions [7, 8]. Kentaro Inamura and Yuichi Ishikawa summarized the characteristic nature of miRNAs which can be used for diagnostics, prognostics, and targeted therapeutics in tumor [9]: (1) the easy availability of miRNAs makes them promising biomarkers for non-invasive liquid biopsy in cancer diagnosis; (2) the stability of miRNAs in formalin-fixed paraffin-embedded (FFPE) samples guarantees the accuracy of measurements using miRNAs which can be performed from FFPE samples collected and preserved in hospitals. Indeed, miRNAs are novel and suitable diagnostic markers to be applied in histological classification or genetic alterations [9].

Recently, increasing studies reporting various expression levels of miRNAs in different cancers (including NSCLC) suggests important roles of certain miRNAs in carcinogenesis [5, 10, 11]. For instance, several miRNAs, such as miRNA-224 and miRNA-30d-5p, have been reported to function either as cancer promoters or as suppressors in NSCLCs [12, 13]. Additionally, previous studies have revealed that the key biological functions of some miRNAs, such as miRNA-107, miRNA-93, and miRNA-148b, are closely associated with clinicopathological variables and to display a diagnostic and prognostic property in NSCLC [14–16]. Even though more and more miRNAs are identified, there are still abundant undegraded miRNAs in tissues as well as in body fluids (e.g., blood, plasma, serum, and sputum) [17, 18]. However, several factors usually give rise to common drawbacks of recent miRNA expression profiling studies resulting from a lack of agreement: inconsistent annotation, application of different technological variations, different methods on data processing and analysis, different sample sizes, and ongoing discovery of novel miRNAs [19, 20].

In order to minimize the drawbacks, meta-analysis approach was applied in this study, which combined several independent studies to increase the statistical power, to identify the key miRNAs in carcinogenesis of NSCLC. Furthermore, we investigated the functions of the target genes and the transcription factors regulated by these miRNAs. Our study provided potential target genes that may be profitable to early detection and precise diagnosis of NSCLC by using miRNAs identified here.

## Methods

### Selection of studies and datasets

NSCLC miRNA expression profiling studies were searched and collected using PubMed databases with terms “non-small cell lung cancer” and “miRNA or microRNA.” Articles for meta-analysis since 2000 were selected as the following criteria. Full text of every study was carefully evaluated. Original experimental literatures that provide detailed miRNA expressions between NSCLC and non-cancerous control in human were selected for further analysis. Expression profile studies of individual preselected candidate genes and studies performed with only cell lines were removed. Studies focusing on different histologic subtypes without using non-cancerous tissue were dislodged as well.

The miRNAs listed by statistically significant expression variations between NSCLC and non-cancerous control in human were extracted from the published papers. Meanwhile, in case the gene list was not available, we also contacted the authors directly. All miRNA names were standardized by miRBase version 21. Many traditional “major” miRNA annotations throughout the main text were re-designated according to miRBase database version 21. Viral non-miRNA and miRNA probes were excluded from the analysis. After the standardization of precursor names, the pre-miRNAs, which are reported in certain studies, were used in the analyses.

### Cluster analysis of datasets

To evaluate the correlations between the outcomes of individual studies, we performed hierarchical cluster analysis. Briefly, overall rank matrix was constructed according to rank matrixes generated from separate analyses for upregulated and downregulated gene with RobustRankAggreg of R package. In the matrix, value 0.5 indicates that this miRNA was not reported in that study, while value above 0.5 indicates that it is upregulated (one minus the normalized rank of miRNA on the upregulated gene lists) and value below 0.5 indicates that this miRNA is downregulated in that study (normalized rank on the downregulated gene lists). Spearman rank correlation with average linkage method was used in cluster analysis.

### Statistical analysis

The extracted miRNAs were prioritized basing on the *p* values in *t* test. *p* < 0.05 was considered as threshold for significance identification. For a better consistence of the identified miRNAs, we applied a novel robust rank aggregation method—RobustRankAggreg of R package [21]. In this method, one *p* value to each element was assigned to indicate how prior it is when compared to a null model. In order to evaluate the stability of acquired *p* values, we left one out cross-validation on the robust rank aggregation algorithm and repeated analyses for 10,000 times. Then, one random gene list was taken out from the analysis for each time. At the end, average of acquired *p* values from each repeat for each miRNA was calculated.

### miRNA target prediction

The potential targets of meta-signature miRNAs were predicted from databases by utilizing target prediction algorithms, TargetScan v6.1 [19]. Considering the experimental threshold of target genes below which protein production is highly repressed together with the prediction algorithms in TargetScan v6.1, the maximum number of target genes for each miRNA with significant expression variation was set to 300. But the other parameters were as default.

### Enrichment analysis

A functional enrichment analysis was performed to examine the enrichment of annotated terms. Gene ontology (GO) enrichment analysis was conducted via using the Database for Annotation Visualization and Integrated Discovery (DAVID). Predicted target genes for each miRNA were used as input and followed by FDR-corrected *p* values (*p* < 0.05) and then visualized as a heatmap. Clustering of the heatmap was according to Pearson correlation and average linkage.

### Transcription factor analysis

The key up- and downregulated miRNAs were uploaded to Tfacts database (http://www.tfacts.org/) [22, 23]. The transcription factors were predicted by *p* value, *q* value, *E* value, and FDR (each value is less than 0.05). The transcription factors regulated by up- and downregulated miRNAs were compared to find out the overlapped ones.

## Results

**Fig. 1:**
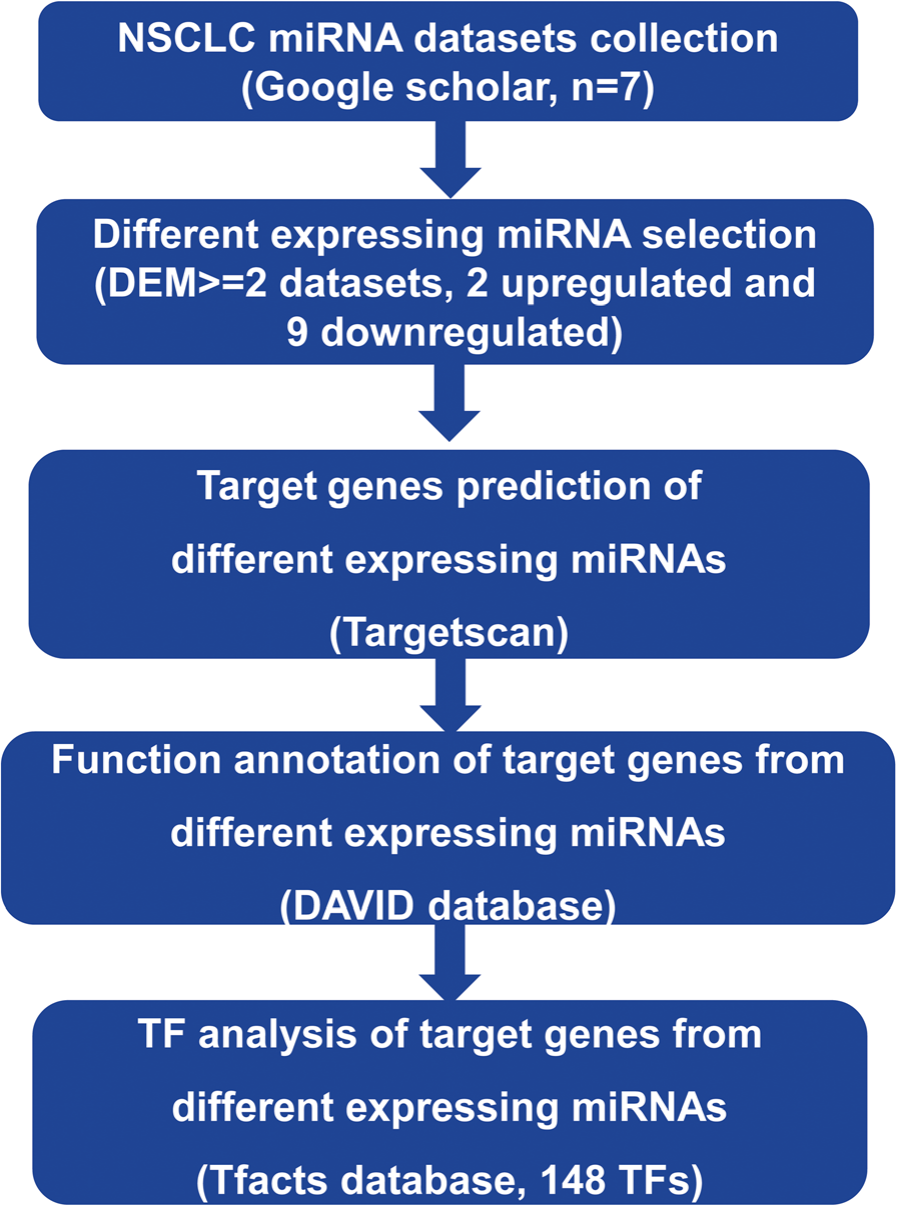
Flow chart of the analysis

Here, we applied meta-analysis to identify the key significantly up- and downregulated miRNAs from the seven available NSCLC miRNA datasets (Table 1) [24–29]. Furthermore, we also predicted potential target genes of the selected miRNAs, of which function annotation and transcription factor (TF) analysis were performed as well (Fig. 1).

**Table 1:** Characteristics of analyzed datasets

Dataset	Samples	Assay type	Validated
1(22)	66 pairs of NSCLC and paracancerous tissues	TaqMan	qRT-PCR
2(23)	335 NSCLC patients, 10 patients	miRCURY LNA Array	qRT-PCR
3(24)	70 pairs of NSCLC and corresponding non-cancerous lung tissues	Agilent	qRT-PCR
4(25)	434 tumor tissues compared with 46 normal lung tissues	TCGA database	qRT-PCR
5(4)	19 NSCLC fresh frozen cancerous and adjacent non-cancerous tissue specimens	xMAP and LNA	qRT-PCR
6(26)	23 patients, tumor and corresponding non-tumor lung tissue	mParaflo microfluidic chip technology	qRT-PCR
7(27)	6 NSCLC tissues and 6 matched normal controls from adjacent tissue	NCBI, GSE29248	–

### Selection of miRNAs from the datasets

**Fig. 2:**
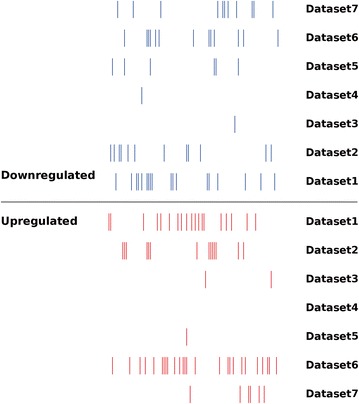
Distribution of tumor-specific miRNA expression changes in NSCLC as reported by primary studies. Short blue and red vertical bars indicate downregulated and upregulated miRNAs, respectively. The *x*-axis listed the miRNA expression profiles from different studies; the *y*-axis displayed databases

According to search criteria, 7 NSCLC miRNA expression profiling datasets from 7 published literatures were gained from public databases. A short description of these studies, such as the assay type, sample numbers, and number of miRNA probes, are provided in Table 1. Although the number of patients investigated was ranging from 6 to 434 across the studies, qRT-PCR was applied on each dataset to verify the reliability of expression difference of miRNAs. Expansion of studied miRnome from 7 datasets was reflected by Scalable Vector Graphics (SVG) of the differentially expressed miRNAs in all datasets. We found that generally, the miRNA expression profiles from different studies had huge variations (Fig. 2).

**Fig. 3:**
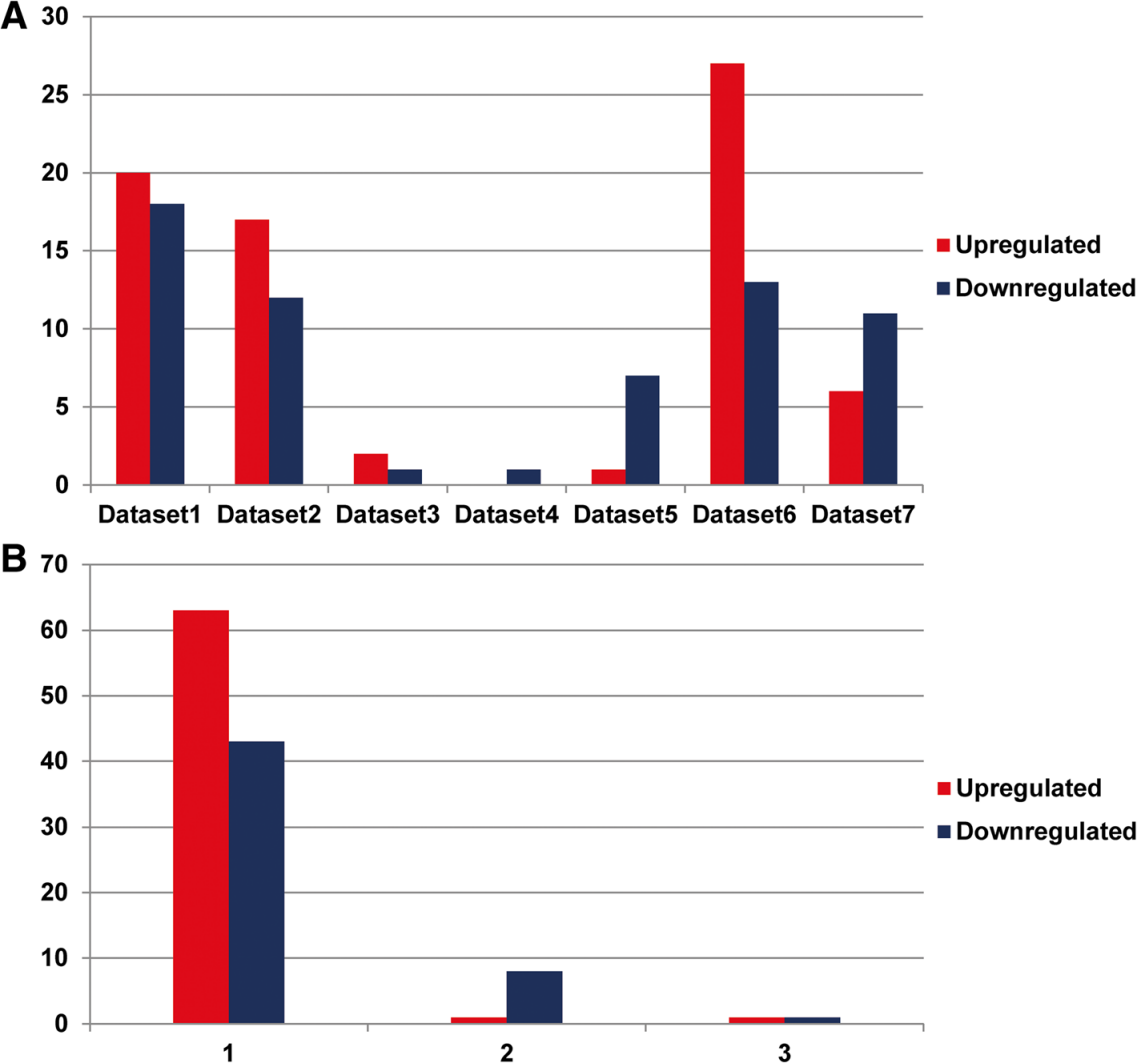
**a** The size of deregulated miRNA varies greatly across the studies. **b** Numbers of deregulated miRNA supported by different number of dataset (*x*-axis). *Y*-axis designates the number of significantly upregulated (blue) or downregulated (red) miRNAs

The size of significantly deregulated miRNA varies largely across the 7 studies. Three datasets showed more than 20 deregulated miRNAs, namely, dataset 1 (38 deregulated miRNAs), dataset 2 (29 deregulated miRNAs), and dataset 6 (40 deregulated miRNAs). Dataset 6 has the most upregulated miRNAs (27 upregulated miRNAs), while dataset 1 has the most downregulated miRNAs (18 downregulated miRNAs) (Fig. 3a).

### NSCLC miRNA meta-signature

In total, based on the 7 independent datasets we selected, 65 (63 in 1 study, 1 and 1 in 2 and 3 study) were reported as significantly upregulated while 52 (42 in 1 study, 9 and 1 in 2 and 3 study) as significantly downregulated (Fig. 3b). However, the dataset-specific upregulated miRNAs were 63 (96.9% of non-redundant upregulated miRNAs) and dataset-specific downregulated miRNAs were 43 (82.7% of non-redundant downregulated miRNAs). The high percentages emphasized the importance of meta-analysis on expression difference of miRNAs in NSCLC. Using robust rank aggregation, we found a statistically significant meta-signature consisting of 2 upregulated and 9 downregulated miRNAs in NSCLC samples compared to non-cancerous tissue (Table 2). These 11 meta-signature miRNAs that displayed highly statistical significance were reported by at least 2 datasets and were considered as key miRNAs in carcinogenesis of NSCLC.

**Table 2:** NSCLC meta-signature miRNAs

miRNA	Chromosome	Beginning	End	Strand	Sequence	Supported datasets
Upregulated						
hsa-miR-21-5p	chr17	59841273	59841294	+	UAGCUUAUCAGACUGAUGUUGA	3
hsa-miR-223-3p	chrX	66018937	66018958	+	UGUCAGUUUGUCAAAUACCCCA	2
Downregulated						
hsa-miR-126-3p	chr9	136670653	136670674	+	UCGUACCGUGAGUAAUAAUGCG	2
hsa-miR-133a-3p	chr18	21825712	21825733	−	UUUGGUCCCCUUCAACCAGCUG	2
hsa-miR-140-5p	chr16	69933103	69933124	+	CAGUGGUUUUACCCUAUGGUAG	2
hsa-miR-143-5p	chr5	149428944	149428965	+	GGUGCAGUGCUGCAUCUCUGGU	2
hsa-miR-145-5p	chr5	149430661	149430683	+	GUCCAGUUUUCCCAGGAAUCCCU	3
hsa-miR-30a-5p	chr6	71403595	71403616	−	UGUAAACAUCCUCGACUGGAAG	2
hsa-miR-30d-3p	chr8	134804879	134804900	−	CUUUCAGUCAGAUGUUUGCUGC	2
hsa-miR-328-3p	chr16	67202327	67202348	−	CUGGCCCUCUCUGCCCUUCCGU	2
hsa-miR-451	chr17	28861403	28861424	−	AAACCGUUACCAUUACUGAGUU	2

According to miRBase, meta-signature miRNA genes were located on different chromosomal locations. Chromosome 5, chromosome 16, and chromosome 17 all harbored 2 different miRNAs; meanwhile, chromosomes 6, 8, 9, and 18 severally harbored one miRNAs (Table 2).

### Target prediction and functional enrichment of meta-signature miRNAs

The high-stringency target gene prediction for meta-signature miRNAs was conducted by TargetScan. We obtained target genes from experimentally supported databases and prediction algorithms. The distribution of target genes is summarized in Additional file 1: Table S1. All the meta-signature miRNAs had the same number of target genes (300). Briefly, 527 non-redundant target genes were affected by the 2 upregulated miRNAs, and 1882 non-redundant target genes were affected by the 9 downregulated genes. The number of target genes affected by each miRNA varied largely. The numbers of target genes affected by 3 miRNAs, namely, hsa-miR-126-3p, hsa-miR-328-3p, and hsa-miR-451, were less than 300, which means these miRNAs may be less conserved in different species [30].

**Fig. 4:**
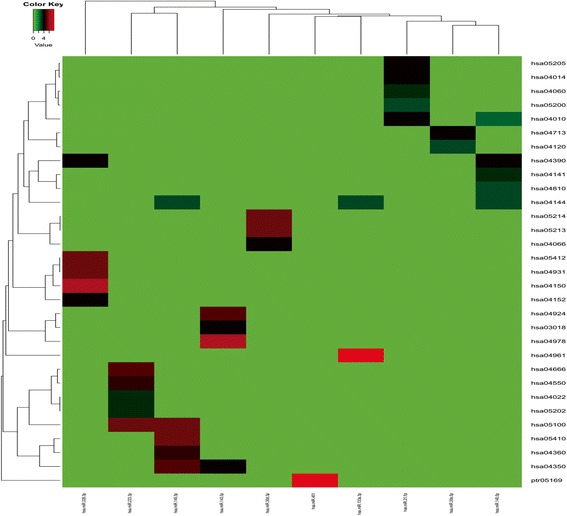
Pathway enrichment of miRNA targets. Pathway enrichment analyses were concordant for all the 7 meta-signature miRNAs. Beside hsa-miR-125b, other 6 meta-signature miRNAs generated rich GO terms. The color from green to red in heatmap represents the increasing GO terms

To elucidate the biological function of meta-signature miRNAs, enrichment analyses were conducted via predicted target genes by using the gene ontology (GO) enrichment analysis and Kyoto Encyclopedia of Genes and Genomes (KEGG) signal pathway analysis on The Database for Annotation, Visualization and Integrated Discovery (DAVID; v6.8). KEGG pathway enrichment analyses were concordant for all the 11 meta-signature miRNAs (Fig. 4). Beside hsa-miR-126-3p, other 10 meta-signature miRNAs generated rich GO terms, especially hsa-miR-21-5p which had the richest GO terms.

Enrichment analysis of the 11 miRNA target sets showed that target genes of upregulated miRNAs were most frequently associated with regulation of transcription from RNA polymerase II promoter, while the targets of downregulated were enriched in positive regulation of transcription from RNA polymerase II promoter as well. Pathways in small GTPase-mediated signal transduction and regulation of branching involved in ureteric bud morphogenesis were also influenced by downregulated miRNAs (Table 3).

**Table 3:** The NSCLC highly saturated pathways by targets of upregulated (red) and downregulated (blue) microRNAs

Term	Count	*p* value	Fold enrichment	Benjamini	FDR
GO:0000122~negative regulation of transcription from RNA polymerase II promoter	56	4.86E−10	2.518139934	1.30E−06	8.63E−07
GO:0045944~positive regulation of transcription from RNA polymerase II promoter	62	9.15E−08	2.06555926	1.22E−04	1.63E−04
GO:0060021~palate development	13	3.25E−06	5.557193787	0.002893	0.005773
GO:0006366~transcription from RNA polymerase II promoter	36	8.39E−06	2.292001365	0.005597	0.014915
GO:0008284~positive regulation of cell proliferation	33	2.87E−05	2.258248746	0.015224	0.050944
GO:0001974~blood vessel remodeling	8	7.27E−05	7.523585434	0.03191	0.129182
GO:0036092~phosphatidylinositol-3-phosphate biosynthetic process	9	1.02E−04	6.045738295	0.038266	0.181279
GO:0006351~transcription, DNA-templated	88	1.35E−04	1.483903889	0.044245	0.24022
GO:0006357~regulation of transcription from RNA polymerase II promoter	29	1.69E−04	2.18433616	0.049011	0.300012
GO:0045944~positive regulation of transcription from RNA polymerase II promoter	151	8.98E−10	1.635197256	4.34E−06	1.70E−06
GO:0045893~positive regulation of transcription, DNA-templated	80	7.28E−06	1.658786271	0.017456	0.013808
GO:0006366~transcription from RNA polymerase II promoter	78	2.54E−05	1.614188342	0.024278	0.048167
GO:0048013~ephrin receptor signaling pathway	22	3.15E−05	2.736997347	0.025043	0.059642
GO:0007264~small GTPase mediated signal transduction	45	2.37E−05	1.941381916	0.028286	0.044989
GO:0090190~positive regulation of branching involved in ureteric bud morphogenesis	10	2.05E−05	5.631142867	0.032546	0.038909
GO:0032331~negative regulation of chondrocyte differentiation	9	6.40E−05	5.664267237	0.043258	0.121289
GO:0007010~cytoskeleton organization	32	7.75E−05	2.126543393	0.045758	0.146799

**Fig. 5:**
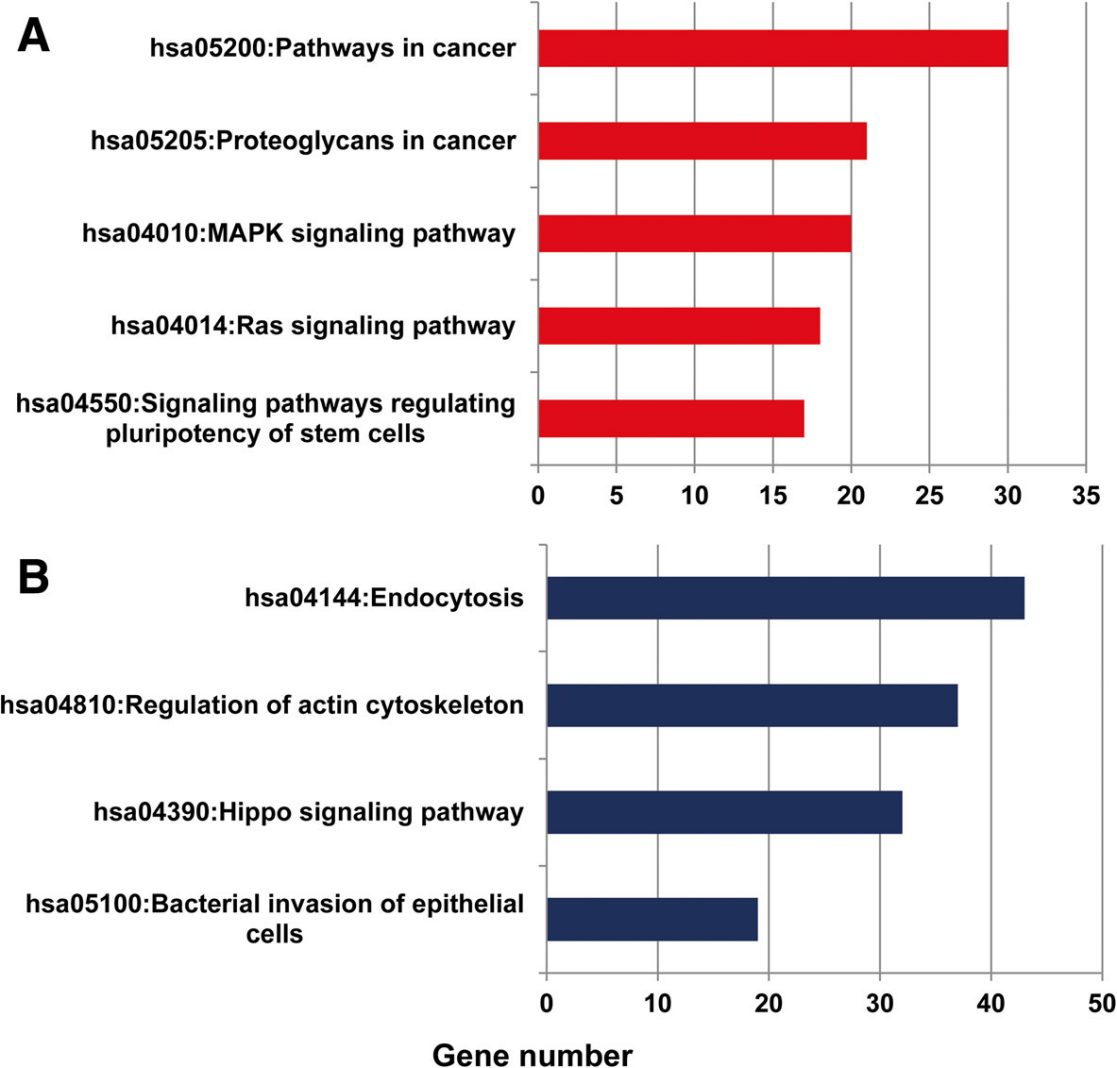
The NSCLC pathway enrichment of target genes of selected microRNAs. **a** Pathways influenced by two upregulated miRNAs. **b** Pathways influenced by nine down-regulated miRNAs

We further analyzed the pathway enrichment of target genes influenced by the miRNAs and found that the target genes regulated by upregulated miRNAs were mainly enriched in pathways in cancer, proteoglycans in cancer, mitogen-activated protein kinases (MAPK) signaling pathway, Ras signaling pathway, and signaling pathways regulating pluripotency of stem cells (Fig. 5a). Additionally, the target genes regulated by downregulated miRNAs were mainly enriched in four groups of pathway, which are endocytosis, actin cytoskeleton, Hippo signaling (Salvador/Warts/Hippo (SWH) pathway), and bacterial invasion of epithelial cells (Fig. 5b).

### Transcription factor analysis

**Fig. 6:**
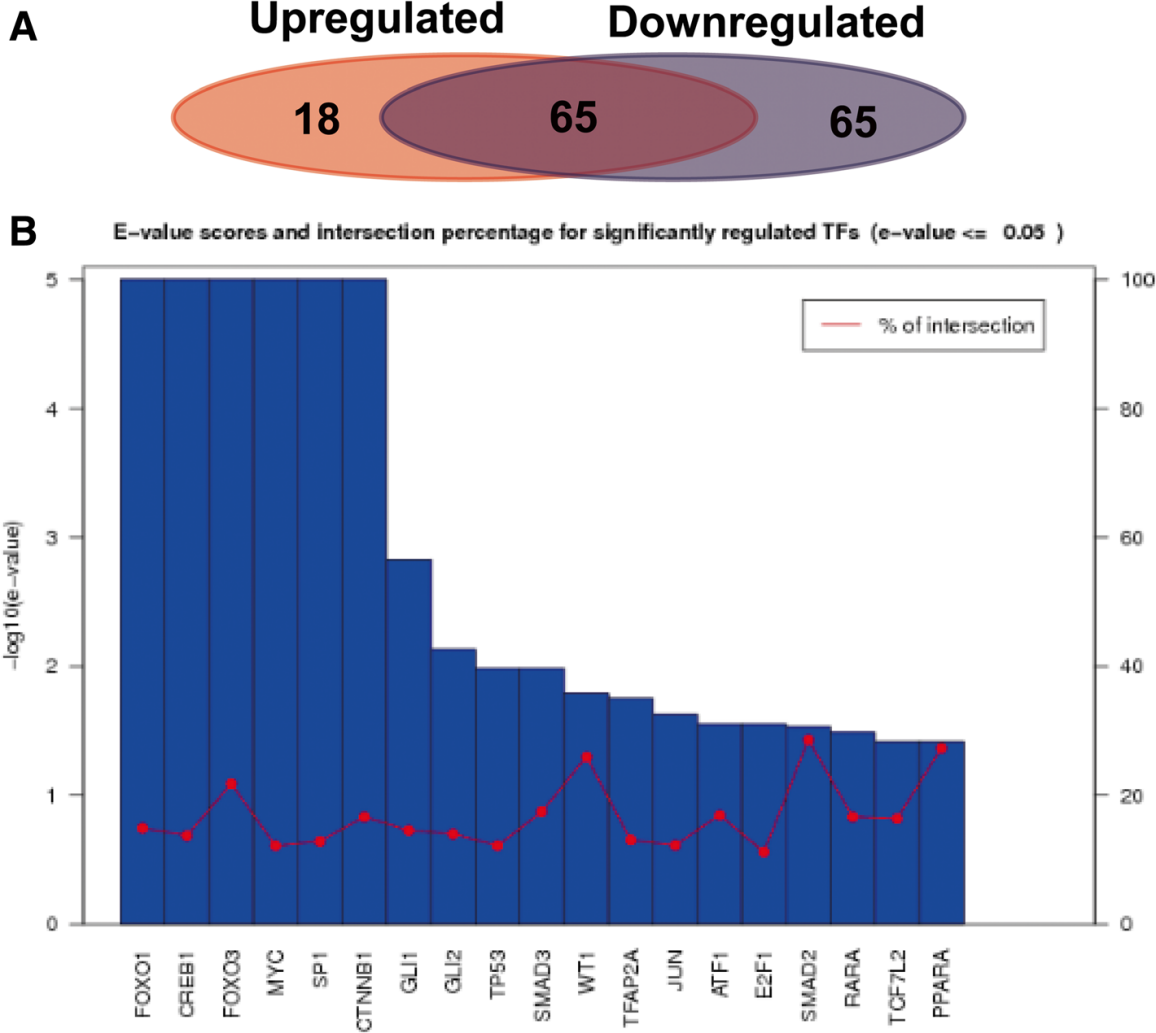
Analysis of transcription factor regulated by miRNAs. **a** Comparison of specific transcription factors influenced by up (blue)- and downregulated (red) miRNAs, and the number of overlapping transcription factors. **b** *E* value and cross-ratio statistics of important transcription factor. *X*-axis listed the names of significantly regulated TFs. *Y*-axis were the *E* value scores in log for the blue bars, the red curve was the intersection percentage of each TF

We further investigated the transcription factors regulated by meta-signature miRNAs and compared the difference of transcription factors between up- and downregulated miRNAs (Additional file 2: Table S2). Transcription factor analysis revealed that 195 interactions were formed between 83 transcription factors and 2 upregulated miRNAs, while 633 interactions were formed between 130 transcription factors and 9 downregulated miRNAs. As shown in the Venn diagram, 65 (43.9%) transcription factors were influenced by both up- and downregulated miRNAs (Fig. 6a). Then, the *E* value scores and intersection percentage for significantly regulated TFs were displayed in detail (Fig. 6b).

## Discussion

Studies analyzing miRNA expressions depending on the evolution of lung cancer have been carried out and reviewed [18]. It is obvious that miRNAs may be potential biomarkers due to their increased specificity and selectivity. However, there is still a need to assign and validate the key miRNAs for different types of lung cancer. In this study, the preferred method we used for gene expression meta-analysis is involved in analysis of raw miRNA expression datasets; however, the unavailability of the raw data often limits the possibility of such rigorous approach. Moreover, changes in the number of miRNAs known at different moment and the technological platform applied in any individual study complicate the integration of raw dataset.

In addition, noisiness of microarray data and the relatively small sample size have caused inconsistency of biological conclusions. Therefore, to get rid of these limitations above, we directly analyzed 7 prioritized miRNA datasets from the 7 published studies and then followed by robust rank aggregation method.

In total, 11 meta-signature miRNAs were identified to play essential roles in carcinogenesis of NSCLC, namely, hsa-miR-21-5p, hsa-miR-223-3p, hsa-miR-126-3p, hsa-miR-140-5p, hsa-miR-133a-3p, hsa-miR-143-5p, hsa-miR-145-5p hsa-miR-30a-5p hsa-miR-30d-3p hsa-miR-328-3p hsa-miR-451, and hsa-miR-140-5p, of which the first 2 showed upregulation and the others displayed downregulated expression. Interestingly, besides the publications we applied in this study, several studies have also reported the importance of these miRNAs not only in studies regarding NSCLC but also in other kinds of cancers. For instance, the overexpression of hsa-miR-21-5p, one of the best studied miRNAs, not only was suggested to be an important prognostic factor for the overall survival of NSCLC patients [31, 32] but also was deregulated in other cancers, such as breast cancer and cervical cancer; it seems to be a common oncogenic miRNA of no tissue specificity [33, 34]. Hsa-miR-126-3p is also reported to display a tumor-suppressive effect on breast cancer cells and be associated with aggressive disease phenotype [35]. Hsa-miR-143-5p exhibits a strong tumor suppressive effect and has been shown to act as anti-oncomirs in gastric cancer and directly target prostaglandin-endoperoxide synthase 2 (COX-2) [36]. Hsa-miR-145-5p also functions as tumor suppressor in gall bladder cancer [37]. Therefore, these meta-signature miRNAs showed great potential to benefit not only NSCLC but also other cancer diagnoses as biomarkers.

The biological function of each meta-signature miRNA was also investigated in our study. One single miRNA may affect expression of multiple target genes; on the other hand, a specific mRNA may be regulated by certain different miRNAs. Subsequently, the miRNAs may induce changes in various processes and pathways and present a further level of mechanism by which NSCLC may be induced. Enriched KEGG showed that target genes of upregulated miRNAs were most frequently associated with regulation of transcription from RNA polymerase II promoter and MAPK pathway. For example, hsa-miR-21, a well-known onco-miRNA, acts on various target genes such as programmed cell death protein 4 (PDCD4) [34] and PTEN [38]. Has-miR-21 has also been reported to influence the tumor formation by regulating Ras-MEK/ERK pathway. The MAPK pathway plays an essential role in lung cancer; the inhibition of MAPK by flavanone was reported to inhibit the metastasis of lung cancer cells [39]. Meanwhile, the targets of downregulated were enriched in positive regulation of transcription from RNA polymerase II promoter, as well as pathways in small GTPase-mediated signal transduction and regulation of branching involved in ureteric bud morphogenesis and Hippo pathway. The Hippo pathway has an important role in tumorigenesis; in mammals, the regulated Hippo pathway can even promote the lung cancer metastasis [40]. In our result, both up- and downregulated miRNAs were found associated with regulation of transcription from the RNA polymerase II promoter. As polymerase II promoter catalyzes the transcription of DNA to synthesize precursors of mRNA and most snRNA and microRNA in the nucleus of eukaryotic cells [41], it is still not easy to draw a conclusion clearly that one of the miRNAs will regulate another one by influencing this promoter. Still, there is a large part of studies regarding the roles of the pathways we identified in NSCLC that needs to be further explored.

Currently, our analysis is restricted to comparison of cancerous and non-cancerous tissue only; however, the 11 most frequently and significantly reported differentially expressed miRNAs could be considered as potential diagnostic or/and prognostic biomarkers. Furthermore, functional enrichment analysis and target predictions may also provide a thread for clarifying the functions of miRNAs in the precise underlying mechanisms and the carcinogenesis of NSCLC. Additionally, from clinical perspectives, sufficient sensitivity and specificity of miRNAs should be further detected in the well-designed clinical studies. Therefore, the findings of this study may provide substantially clinical implications or significance.

## Conclusions

In conclusion, we utilized a meta-analysis method to critically converge and dissect the heterogeneous miRNA expression profiling datasets regarding NSCLC. In total, 11 meta-signature miRNAs were identified, which are consistently up- and downregulated, and highly significant across 7 different studies. Due to the development of miRNA-based tests, our analysis achieves certain highlights on identifying new biomarkers for NSCLC, but still, it is in need that rigorous evaluation of the results should be carried out prior to proceeding to clinical treatments.

## Additional files

Additional file 1: Table S1. NSCLC miRNA targets. (XLSX 296 kb)

Additional file 2: Table S2. TF list of miRNA targets. (XLSX 33 kb)
